# 3-Anilinomethyl-5-chloro-1,3-­benzoxazol-2(3*H*)-one

**DOI:** 10.1107/S1600536812017709

**Published:** 2012-04-28

**Authors:** Abdullah Aydın, Zeynep Soyer, Mehmet Akkurt, Orhan Büyükgüngör

**Affiliations:** a Department of Science Education, Faculty of Education, Kastamonu University, 37200 Kastamonu, Turkey; b Department of Pharmaceutical Chemistry, Faculty of Pharmacy, Ege University, 35100 Ízmir, Turkey; c Department of Physics, Faculty of Sciences, Erciyes University, 38039 Kayseri, Turkey; d Department of Physics, Faculty of Arts and Sciences, Ondokuz Mayıs University, 55139 Samsun, Turkey

## Abstract

In the title compound, C_14_H_11_ClN_2_O_2_, the 2,3-dihydro-1,3-benzoxazole ring system is essentially planar [maximum deviation = 0.009 (2) Å] and makes a dihedral angle of 79.15 (7)° with the phenyl ring. Inter­molecular N—H⋯O and weak C—H⋯Cl hydrogen bonds occur in the crystal structure.

## Related literature

For the synthesis and biological activity of compounds with a benzoxazolone nucleus, see; Varma & Nobles (1968 ▶); Courtois *et al.* (2004 ▶); Deng *et al.* (2006 ▶); Ivanova *et al.* (2007 ▶); Koksal *et al.* (2002 ▶, 2005 ▶); Onkol *et al.* (2001 ▶); Soyer *et al.* (2005 ▶); Ucar *et al.* (1998 ▶); Unlu *et al.* (2003 ▶). For bond-length data, see: Allen *et al.* (1987 ▶). For a related structure, see: Aydın *et al.* (2004 ▶).
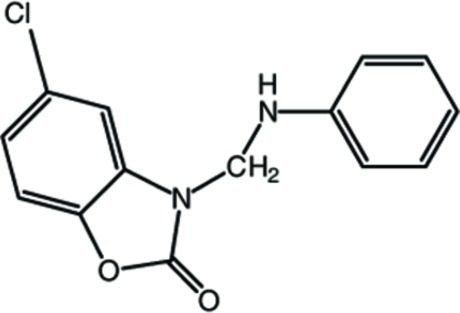


## Experimental

### 

#### Crystal data

C_14_H_11_ClN_2_O_2_
*M* *_r_* = 274.70
Monoclinic, 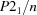

*a* = 9.7379 (5) Å
*b* = 12.4797 (7) Å
*c* = 10.2392 (7) Å
β = 93.129 (5)°
*V* = 1242.48 (13) Å^3^
*Z* = 4
Mo *K*α radiation
μ = 0.31 mm^−1^
*T* = 296 K
0.80 × 0.48 × 0.26 mm

#### Data collection

Stoe IPDS 2 diffractometer
Absorption correction: integration (*X-RED32*; Stoe & Cie, 2002 ▶) *T*_min_ = 0.837, *T*_max_ = 0.924
20574 measured reflections
3051 independent reflections
2544 reflections with *I* > 2σ(*I*)
*R*_int_ = 0.046

#### Refinement

*R*[*F* ^2^ > 2σ(*F* ^2^)] = 0.043
*wR*(*F* ^2^) = 0.106
*S* = 1.07
3051 reflections
172 parameters
H-atom parameters constrained
Δρ_max_ = 0.22 e Å^−3^
Δρ_min_ = −0.22 e Å^−3^

### 

Data collection: *X-AREA* (Stoe & Cie, 2002 ▶); cell refinement: *X-AREA*; data reduction: *X-RED32* (Stoe & Cie, 2002 ▶); program(s) used to solve structure: *SHELXS97* (Sheldrick, 2008 ▶); program(s) used to refine structure: *SHELXL97* (Sheldrick, 2008 ▶); molecular graphics: *ORTEP-3 for Windows* (Farrugia, 1997 ▶); software used to prepare material for publication: *WinGX* (Farrugia, 1999 ▶) and *PLATON* (Spek, 2009 ▶).

## Supplementary Material

Crystal structure: contains datablock(s) global, I. DOI: 10.1107/S1600536812017709/xu5511sup1.cif

Structure factors: contains datablock(s) I. DOI: 10.1107/S1600536812017709/xu5511Isup2.hkl

Supplementary material file. DOI: 10.1107/S1600536812017709/xu5511Isup3.cml

Additional supplementary materials:  crystallographic information; 3D view; checkCIF report

## Figures and Tables

**Table 1:** Hydrogen-bond geometry (Å, °)

*D*—H⋯*A*	*D*—H	H⋯*A*	*D*⋯*A*	*D*—H⋯*A*
N2—H2*A*⋯O2^i^	0.86	2.30	3.1296 (17)	162
C11—H11⋯Cl1^ii^	0.93	2.70	3.5295 (17)	150

Symmetry codes: (i) 
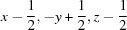
; (ii) 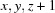.

**Fig. 1.**
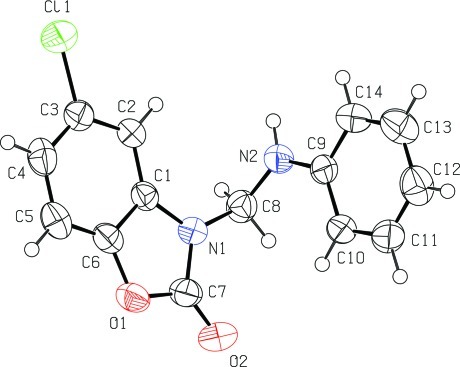
The molecule shown with the atom numbering scheme. Displacement ellipsoids for non-H atoms are drawn at the 50% probability level.

**Fig. 2.**
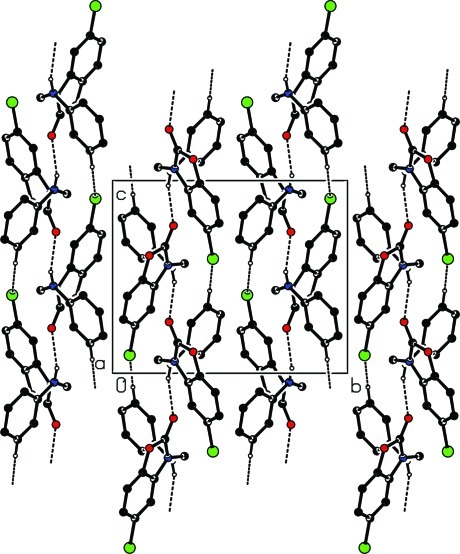
The packing and hydrogen bonding of (I) viewed down the a axis. H atoms not involved in hydrogen bondings are omitted for the sake of clarity.

## supplementary crystallographic information

### Comment

The benzoxazolone nucleus represents an important pharmacophore present in pharmaceutical products. The compounds possessing this structure have a broad spectrum of biological activities, such as anti-HIV (Deng *et al.*, 2006), anticancer (Ivanova *et al.*, 2007), analgesic (Unlu *et al.*, 2003), anti-inflammatory (Koksal *et al.*, 2005), antinociceptive (Onkol *et al.*, 2001), antimicrobial (Koksal *et al.*, 2002), anticonvulsant (Ucar *et al.*, 1998), antimalarial (Courtois *et al.*, 2004), human leukocyte MPO clorinating inhibitor activity (Soyer *et al.*, 2005).

In addition to, this compound was synthesized before by (Varma & Nobles, 1968) and they reported that most benzoxazolinone compounds have shown significant antibacterial activity.

In the title compound (I), (Fig. 1), the mean planes of the 2,3-dihydro-1,3-benzoxazole and phenyl rings make a dihedral angle of 79.15 (7)° with each other. The N1—C8—N2—C9 torsion angle is -72.99 (18)°. The bond lengths in (I) are normal and correspond to those observed in the related compound (Allen *et al.*, 1987).

The Cl1—C3 and N1—C1 bond lengths are 1.7315 (16) Å, and 1.3914 (18) Å, respectively. The Cl1—C3—C4 and O2—C7—N1 bond angles are 118.73 (13) ° and 129.27 (15) °, respectively. The bond lengths and the bond angles of (I) are comparable to those observed in related structure (Aydın *et al.*, 2004).

The crystal structure is stabilized by intermolecular N—H···O and C—H···Cl interactions (Table 1 and Fig. 2), connecting the molecules along the [001] direction.

### Experimental

4-Chloro-2-aminophenol (10 mmol), urea (50 mmol) and 37% HCl (2.5 ml) were irradiated (300 W, 413 K) for 15 min in a microwave oven. After completion of reaction (by monitoring with TLC), water (10 ml) was added to the reaction mixture and stirred at room temperature for 1 h. The resulting precipitate was filtered and washed with water. The crude product crystallized from ethanol-water (1:1) to yield 5-chloro-2(3*H*)-benzoxazolone. This compound (2 mmol) was dissolved in methanol (5 ml). Aniline (2 mmol) and 37% formalin (2.5 mmol) were added to this solution. The mixture was stirred vigorously for 3 h. The resulting precipitate was filtered and washed with cold methanol. The crude product was crystallized from methanol.

M.p.: 463 K. Yield 84%; IR *v*_max_ (FTIR/ATR): 3398, 3066, 1750, 1604 cm^-1^; ^1^H-NMR (DMSO-d_6_): δ 5.23 (2*H*, d, J=7.0 Hz, CH_2_), 6.61(1*H*, t, J=7.4 Hz, H-Aniline), 6.73-6.75 (2*H*, m, H-Aniline), 6.96 (1*H*, t, J=7.3 Hz, NH), 7.09 (2*H*, t, J=7.4 Hz, H-Aniline) 7.14 (1*H*, dd, J=2.3; 8.6 Hz, H-Benzoxazolone), 7.31 (1*H*, d, J=8.6 Hz, H-Benzoxazolone), 7.70 (1*H*, d, J=2.3 Hz, H-Benzoxazolone) p.p.m.; MS (ESI) m/*z* (%): 275 (*M*+H, 11), 277 (*M*+H+2, 4).

### Refinement

H atoms were positioned geometrically and refined using a riding model with N—H = 0.86 Å, C—H = 0.93 and 0.97 Å, and *U*_iso_(H) = 1.2*U*_eq_(C,N).

### Crystal data

**Table:**

C_14_H_11_ClN_2_O_2_	*F*(000) = 568
*M**_r_* = 274.70	*D*_x_ = 1.469 Mg m^−^^3^
Monoclinic, *P*2_1_/*n*	Mo *K*α radiation, λ = 0.71073 Å
Hall symbol: -P 2yn	Cell parameters from 27572 reflections
*a* = 9.7379 (5) Å	θ = 2.1–28.6°
*b* = 12.4797 (7) Å	µ = 0.31 mm^−^^1^
*c* = 10.2392 (7) Å	*T* = 296 K
β = 93.129 (5)°	Prism, colourless
*V* = 1242.48 (13) Å^3^	0.80 × 0.48 × 0.26 mm
*Z* = 4	

### Data collection

**Table:**

Stoe IPDS 2 diffractometer	3051 independent reflections
Radiation source: sealed X-ray tube, 12 x 0.4 mm long-fine focus	2544 reflections with *I* > 2σ(*I*)
Plane graphite monochromator	*R*_int_ = 0.046
Detector resolution: 6.67 pixels mm^-1^	θ_max_ = 28.2°, θ_min_ = 2.6°
ω scans	*h* = −12→12
Absorption correction: integration (*X-RED32*; Stoe & Cie, 2002)	*k* = −16→16
*T*_min_ = 0.837, *T*_max_ = 0.924	*l* = −13→13
20574 measured reflections	

### Refinement

**Table:**

Refinement on *F*^2^	Primary atom site location: structure-invariant direct methods
Least-squares matrix: full	Secondary atom site location: difference Fourier map
*R*[*F*^2^ > 2σ(*F*^2^)] = 0.043	Hydrogen site location: inferred from neighbouring sites
*wR*(*F*^2^) = 0.106	H-atom parameters constrained
*S* = 1.07	*w* = 1/[σ^2^(*F*_o_^2^) + (0.0456*P*)^2^ + 0.2748*P*] where *P* = (*F*_o_^2^ + 2*F*_c_^2^)/3
3051 reflections	(Δ/σ)_max_ < 0.001
172 parameters	Δρ_max_ = 0.22 e Å^−^^3^
0 restraints	Δρ_min_ = −0.22 e Å^−^^3^

### Special details

**Table:**

Geometry. Bond distances, angles *etc*. have been calculated using the rounded fractional coordinates. All su's are estimated from the variances of the (full) variance-covariance matrix. The cell e.s.d.'s are taken into account in the estimation of distances, angles and torsion angles
Refinement. Refinement on *F*^2^ for ALL reflections except those flagged by the user for potential systematic errors. Weighted *R*-factors *wR* and all goodnesses of fit *S* are based on *F*^2^, conventional *R*-factors *R* are based on *F*, with *F* set to zero for negative *F*^2^. The observed criterion of *F*^2^ > σ(*F*^2^) is used only for calculating -*R*-factor-obs *etc*. and is not relevant to the choice of reflections for refinement. *R*-factors based on *F*^2^ are statistically about twice as large as those based on *F*, and *R*-factors based on ALL data will be even larger.

### Fractional atomic coordinates and isotropic or equivalent isotropic displacement parameters (Å^2^)

**Table:**

	*x*	*y*	*z*	*U*_iso_*/*U*_eq_	
Cl1	0.37199 (5)	0.42730 (6)	−0.40607 (5)	0.0887 (2)	
O1	0.61747 (10)	0.34298 (10)	0.11087 (11)	0.0535 (3)	
O2	0.52561 (13)	0.24080 (12)	0.26644 (11)	0.0679 (4)	
N1	0.41738 (11)	0.26396 (10)	0.06103 (11)	0.0428 (4)	
N2	0.16989 (12)	0.24829 (12)	0.04683 (12)	0.0508 (4)	
C1	0.45104 (13)	0.32285 (11)	−0.04809 (13)	0.0392 (4)	
C2	0.38425 (14)	0.33757 (13)	−0.16827 (14)	0.0454 (4)	
C3	0.45056 (16)	0.40463 (14)	−0.25277 (15)	0.0520 (5)	
C4	0.57449 (16)	0.45354 (13)	−0.22095 (18)	0.0553 (5)	
C5	0.64095 (15)	0.43708 (13)	−0.09935 (17)	0.0539 (5)	
C6	0.57568 (14)	0.37153 (12)	−0.01549 (15)	0.0447 (4)	
C7	0.51813 (15)	0.27708 (13)	0.15798 (15)	0.0495 (5)	
C8	0.29647 (15)	0.19414 (13)	0.06944 (15)	0.0479 (5)	
C9	0.11468 (13)	0.31842 (12)	0.13447 (13)	0.0422 (4)	
C10	0.17189 (15)	0.33453 (13)	0.26004 (14)	0.0475 (4)	
C11	0.10954 (17)	0.40404 (15)	0.34387 (16)	0.0565 (5)	
C12	−0.00743 (18)	0.45947 (15)	0.30515 (19)	0.0624 (6)	
C13	−0.06427 (19)	0.44406 (16)	0.1809 (2)	0.0659 (6)	
C14	−0.00497 (17)	0.37461 (15)	0.09612 (16)	0.0567 (5)	
H2	0.30060	0.30500	−0.19140	0.0550*	
H2A	0.12430	0.23690	−0.02610	0.0610*	
H4	0.61400	0.49800	−0.28140	0.0660*	
H5	0.72520	0.46880	−0.07610	0.0650*	
H8A	0.30210	0.13680	0.00590	0.0580*	
H8B	0.29860	0.16180	0.15570	0.0580*	
H10	0.25200	0.29870	0.28780	0.0570*	
H11	0.14770	0.41340	0.42830	0.0680*	
H12	−0.04750	0.50670	0.36210	0.0750*	
H13	−0.14370	0.48100	0.15370	0.0790*	
H14	−0.04490	0.36490	0.01240	0.0680*	

### Atomic displacement parameters (Å^2^)

**Table:**

	*U*^11^	*U*^22^	*U*^33^	*U*^12^	*U*^13^	*U*^23^
Cl1	0.0635 (3)	0.1420 (6)	0.0602 (3)	0.0059 (3)	−0.0005 (2)	0.0431 (3)
O1	0.0397 (5)	0.0669 (7)	0.0528 (6)	−0.0022 (5)	−0.0074 (4)	−0.0061 (5)
O2	0.0674 (8)	0.0890 (9)	0.0457 (6)	0.0034 (7)	−0.0112 (5)	0.0084 (6)
N1	0.0373 (6)	0.0508 (7)	0.0399 (6)	−0.0004 (5)	−0.0007 (4)	0.0013 (5)
N2	0.0387 (6)	0.0736 (9)	0.0397 (6)	−0.0032 (6)	−0.0006 (5)	−0.0068 (6)
C1	0.0332 (6)	0.0416 (7)	0.0429 (7)	0.0030 (5)	0.0039 (5)	−0.0028 (6)
C2	0.0337 (6)	0.0572 (9)	0.0452 (7)	0.0012 (6)	0.0011 (5)	0.0028 (6)
C3	0.0433 (7)	0.0645 (10)	0.0486 (8)	0.0091 (7)	0.0050 (6)	0.0111 (7)
C4	0.0468 (8)	0.0528 (9)	0.0678 (10)	0.0010 (7)	0.0166 (7)	0.0096 (8)
C5	0.0391 (7)	0.0515 (9)	0.0716 (10)	−0.0055 (6)	0.0082 (7)	−0.0054 (8)
C6	0.0353 (6)	0.0478 (8)	0.0507 (8)	0.0027 (6)	0.0002 (6)	−0.0068 (6)
C7	0.0431 (7)	0.0582 (9)	0.0464 (8)	0.0066 (7)	−0.0044 (6)	−0.0024 (7)
C8	0.0477 (8)	0.0487 (8)	0.0476 (8)	−0.0050 (6)	0.0049 (6)	0.0000 (6)
C9	0.0365 (6)	0.0503 (8)	0.0400 (7)	−0.0100 (6)	0.0033 (5)	0.0036 (6)
C10	0.0385 (7)	0.0609 (9)	0.0427 (7)	−0.0069 (6)	−0.0008 (5)	0.0020 (7)
C11	0.0523 (9)	0.0708 (11)	0.0463 (8)	−0.0130 (8)	0.0010 (6)	−0.0104 (8)
C12	0.0565 (9)	0.0612 (10)	0.0704 (11)	−0.0046 (8)	0.0110 (8)	−0.0156 (9)
C13	0.0523 (9)	0.0667 (11)	0.0778 (12)	0.0099 (8)	−0.0039 (8)	−0.0028 (9)
C14	0.0493 (8)	0.0695 (11)	0.0501 (8)	0.0016 (7)	−0.0081 (7)	0.0007 (8)

### Geometric parameters (Å, º)

**Table:**

Cl1—C3	1.7315 (16)	C9—C14	1.398 (2)
O1—C6	1.3817 (19)	C9—C10	1.388 (2)
O1—C7	1.3769 (19)	C10—C11	1.384 (2)
O2—C7	1.198 (2)	C11—C12	1.373 (2)
N1—C1	1.3914 (18)	C12—C13	1.373 (3)
N1—C7	1.3669 (19)	C13—C14	1.376 (3)
N1—C8	1.4711 (19)	C2—H2	0.9300
N2—C8	1.414 (2)	C4—H4	0.9300
N2—C9	1.3831 (19)	C5—H5	0.9300
N2—H2A	0.8600	C8—H8A	0.9700
C1—C2	1.3725 (19)	C8—H8B	0.9700
C1—C6	1.3818 (19)	C10—H10	0.9300
C2—C3	1.388 (2)	C11—H11	0.9300
C3—C4	1.376 (2)	C12—H12	0.9300
C4—C5	1.387 (2)	C13—H13	0.9300
C5—C6	1.368 (2)	C14—H14	0.9300
			
C6—O1—C7	107.78 (11)	C9—C10—C11	119.87 (14)
C1—N1—C7	109.19 (11)	C10—C11—C12	121.47 (16)
C1—N1—C8	125.75 (11)	C11—C12—C13	118.90 (17)
C7—N1—C8	124.99 (12)	C12—C13—C14	120.73 (17)
C8—N2—C9	124.23 (12)	C9—C14—C13	120.73 (15)
C9—N2—H2A	118.00	C1—C2—H2	122.00
C8—N2—H2A	118.00	C3—C2—H2	122.00
N1—C1—C6	106.29 (12)	C3—C4—H4	120.00
N1—C1—C2	132.05 (12)	C5—C4—H4	120.00
C2—C1—C6	121.65 (13)	C4—C5—H5	122.00
C1—C2—C3	115.20 (13)	C6—C5—H5	122.00
Cl1—C3—C4	118.73 (13)	N1—C8—H8A	109.00
Cl1—C3—C2	117.77 (12)	N1—C8—H8B	109.00
C2—C3—C4	123.49 (15)	N2—C8—H8A	109.00
C3—C4—C5	120.48 (15)	N2—C8—H8B	109.00
C4—C5—C6	116.25 (14)	H8A—C8—H8B	108.00
C1—C6—C5	122.93 (14)	C9—C10—H10	120.00
O1—C6—C5	128.24 (13)	C11—C10—H10	120.00
O1—C6—C1	108.83 (12)	C10—C11—H11	119.00
O1—C7—N1	107.90 (12)	C12—C11—H11	119.00
O1—C7—O2	122.84 (14)	C11—C12—H12	121.00
O2—C7—N1	129.27 (15)	C13—C12—H12	121.00
N1—C8—N2	113.63 (13)	C12—C13—H13	120.00
N2—C9—C10	122.83 (13)	C14—C13—H13	120.00
C10—C9—C14	118.29 (14)	C9—C14—H14	120.00
N2—C9—C14	118.87 (13)	C13—C14—H14	120.00
			
C7—O1—C6—C5	179.00 (16)	N1—C1—C2—C3	179.08 (15)
C6—O1—C7—O2	−178.94 (16)	C6—C1—C2—C3	−0.3 (2)
C7—O1—C6—C1	−0.57 (16)	N1—C1—C6—O1	0.04 (16)
C6—O1—C7—N1	0.88 (16)	C2—C1—C6—C5	0.0 (2)
C7—N1—C1—C2	−178.96 (16)	C1—C2—C3—Cl1	−179.79 (12)
C8—N1—C1—C2	3.9 (2)	C1—C2—C3—C4	0.2 (2)
C1—N1—C7—O1	−0.86 (16)	C2—C3—C4—C5	0.3 (3)
C8—N1—C7—O1	176.31 (13)	Cl1—C3—C4—C5	−179.75 (13)
C1—N1—C7—O2	178.93 (17)	C3—C4—C5—C6	−0.6 (2)
C8—N1—C7—O2	−3.9 (3)	C4—C5—C6—C1	0.5 (2)
C1—N1—C8—N2	−59.57 (18)	C4—C5—C6—O1	−179.03 (15)
C7—N1—C8—N2	123.72 (15)	N2—C9—C10—C11	−178.47 (15)
C8—N1—C1—C6	−176.64 (13)	C14—C9—C10—C11	0.6 (2)
C7—N1—C1—C6	0.51 (16)	N2—C9—C14—C13	179.13 (16)
C8—N2—C9—C10	−6.8 (2)	C10—C9—C14—C13	0.0 (2)
C9—N2—C8—N1	−72.99 (18)	C9—C10—C11—C12	−1.1 (3)
C8—N2—C9—C14	174.17 (15)	C10—C11—C12—C13	0.9 (3)
C2—C1—C6—O1	179.57 (13)	C11—C12—C13—C14	−0.3 (3)
N1—C1—C6—C5	−179.55 (14)	C12—C13—C14—C9	−0.2 (3)

### Hydrogen-bond geometry (Å, º)

**Table:**

*D*—H···*A*	*D*—H	H···*A*	*D*···*A*	*D*—H···*A*
N2—H2*A*···O2^i^	0.86	2.30	3.1296 (17)	162
C11—H11···Cl1^ii^	0.93	2.70	3.5295 (17)	150

Symmetry codes: (i) *x*−1/2, −*y*+1/2, *z*−1/2; (ii) *x*, *y*, *z*+1.
